# Valorization of Agricultural Waste Lignocellulosic Fibers for Poly(3-Hydroxybutyrate-Co-Valerate)-Based Composites in Short Shelf-Life Applications

**DOI:** 10.3390/polym15234507

**Published:** 2023-11-23

**Authors:** Kerly Samaniego-Aguilar, Estefanía Sánchez-Safont, Andreina Rodríguez, Anna Marín, María V. Candal, Luis Cabedo, Jose Gamez-Perez

**Affiliations:** 1 Polymers and Advanced Materials Group (PIMA), Universitat Jaume I, Av. Sos Baynat s/n, 12071 Castelló, Spain; samanieg@uji.es (K.S.-A.); esafont@uji.es (E.S.-S.); escala@uji.es (A.R.); anmarin@uji.es (A.M.); lcabedo@uji.es (L.C.); 2 CEBIMAT Lab S.L., Universitat Jaume I, Av. Sos Baynat s/n, 12071 Castelló, Spain; 3 School of Engineering, Science and Technology, Valencian International University (VIU), 46002 Valencia, Spain; mariavirginiacandal@campusviu.es

**Keywords:** polyhydroxybutyrate-co-valerate, biocircularity, biobased hybrid composite, lignocellulosic fibers, mechanical performance, biodisintegration, thermoforming

## Abstract

Biocircularity could play a key role in the circular economy, particularly in applications where organic recycling (composting) has the potential to become a preferred waste management option, such as food packaging. The development of fully biobased and biodegradable composites could help reduce plastic waste and valorize agro-based residues. In this study, extruded films made of composites of polyhydroxybutyrate-co-valerate (PHBV) and lignocellulosic fibers, namely almond shell (AS) and Oryzite^®^ (OR), a polymer hybrid composite precursor, have been investigated. Scanning electron microscopy (SEM) analysis revealed a weak fiber–matrix interfacial interaction, although OR composites present a better distribution of the fiber and a virtually lower presence of “pull-out”. Thermogravimetric analysis showed that the presence of fibers reduced the onset and maximum degradation temperatures of PHBV, with a greater reduction observed with higher fiber content. The addition of fibers also affected the melting behavior and crystallinity of PHBV, particularly with OR addition, showing a decrease in crystallinity, melting, and crystallization temperatures as fiber content increased. The mechanical behavior of composites varied with fiber type and concentration. While the incorporation of AS results in a reduction in all mechanical parameters, the addition of OR leads to a slight improvement in elongation at break. The addition of fibers improved the thermoformability of PHBV. In the case of AS, the improvement in the processing window was achieved at lower fiber contents, while in the case of OR, the improvement was observed at a fiber content of 20%. Biodisintegration tests showed that the presence of fibers promoted the degradation of the composites, with higher fiber concentrations leading to faster degradation. Indeed, the time of complete biodisintegration was reduced by approximately 30% in the composites with 20% and 30% AS.

## 1. Introduction

The current consumption patterns within a linear economic system promote an environmentally unsustainable situation. This encompasses concerns related to the exploitation of resources and energy consumption, as well as the generation of emissions and the accumulation of large volumes of waste. One of the key factors in reversing this situation is to achieve a real transition towards a circular economy [1,2]. Circularity is based on the minimization of waste through reduction, reuse, and recycling whenever possible to create added value. In this regard, plastic waste represents a major challenge [3]. In the paradigm of the circular economy, there is no single preferred solution for the management of plastic waste. Instead, a variety of approaches are proposed, taking into account the specific limitations associated with each method. In this regard, organic recycling emerges as a viable alternative for products where mechanical or chemical recycling may not be practical or cost-effective within the existing waste management framework. Examples of such products include multilayered packaging composed of different polymers in each layer, very small or lightweight packaging, and items that are typically heavily contaminated by food residues, such as single-use mayonnaise containers or candy wrappers. Those contaminants and non-uniformity of the plastic stream are severe drawbacks for other processes like pyrolysis [4] or hydrogenation [5], which are more suitable for other sources of plastic waste. Additionally, small disposable plastic items like stirrers and certain plastic components used in agri-food applications, such as labels, rubber strips, and threads, also fall into this category. Given their characteristics, these products are generally non-recyclable and offer limited options for circularity, making organic treatment a logical choice.

In Europe, approximately 30 million tons of post-consumer plastic were collected in 2021, mostly from packaging applications (61%). According to the latest published reports, current recycling rates reach figures close to 35%. However, 65% of plastic waste is still not valorized in the circular economy [6]. Furthermore, while the precise quantity of plastic released into the environment in an uncontrolled manner remains unknown, research suggests that between 10 and 20 million tons of plastic enter the oceans annually. Due to their low biodegradability, conventional plastics contribute to the accumulation of plastic waste in the environment. This poses significant risks to wildlife and ecosystems. Despite their slow degradation, environmental conditions can lead to the breakdown of these plastics into smaller microplastics, which can be harmful to both marine and terrestrial environments. These microplastics persist in the environment, causing long-term ecological damage [7,8,9,10]. Additionally, it is estimated that plastic production and incineration released 850 million tons of greenhouse gases into the atmosphere in 2019, and projections indicate that, if current conditions persist, this figure could rise to 2.8 trillion tons by 2050 [11].

These figures could be improved by increasing recycling rates. However, conventional recycling results are particularly difficult in certain applications, such as food packaging. The main drawbacks lie in the difficulty of separating their components and ensuring the optimal quality of the recycled material. In this scenario, the substitution of conventional plastics with biodegradable alternatives could be a significant contribution to mitigating the issue of plastic waste, especially in applications where organic recycling (composting) can be considered a viable option within a biocircular context [12].

The use of some synthetic linear polyesters, which are known to be biodegradable, such as poly lactic acid (PLA), polybutylene adipate-co-terephthalate (PBAT), or polybutylene succinate (PBS), has been proposed for applications where the end-of-life is compatible with industrial composting [13,14,15]. In this sense, polyhydroxyalkanoates (PHA), a family of bacterial biopolyesters, show significant potential for integration into biocircular economy circuits [16]. Among them, polyhydroxybutyrate-co-valerate (PHBV) has demonstrated biodegradability in various environments, such as composting, soil, and marine conditions [17]. PHBV is biocompatible with living organisms and poses no toxicity risks when ingested [18]. Furthermore, PHBV exhibits comparable physical, thermal, and mechanical properties to conventional plastics like polypropylene (PP) and offers barrier properties similar to polyethylene terephthalate (PET). It can be processed using conventional techniques such as extrusion, injection molding, film blowing, and thermoforming [18,19]. However, there are still limitations hindering its practical industrial application. These include its high sensitivity to thermal degradation, resulting in a narrow processing window, low toughness, and, most importantly, high production costs [20,21,22,23].

One common approach to overcoming cost limitations is the incorporation of a cheaper secondary phase to create a composite material. The utilization of vegetal lignocellulosic fibers as fillers offers several advantages, including their low density, good thermal properties, high hardness, abundant availability, biodegradability, and low cost [24,25,26]. Therefore, the development of such composites not only contributes to the overall cost reduction but also provides an opportunity for the valorization of agricultural residues, favoring economic circularity. In fact, approximately 140 billion tons of lignocellulosic waste are generated globally every year. However, managing and utilizing this waste remains a challenge for the agricultural industry. Currently, these residues are often buried, incinerated, or dumped in landfills, causing adverse environmental impacts. In developing countries, the burning of agricultural residues contributes significantly to air pollution, releasing greenhouse gases and other harmful substances. A recent life cycle analysis study of biocomposites based on PHBV and lignocellulosic fibers (vine shoots) concludes that both the price and the impact on global warming can be reduced by 25% and 20%, respectively, with a 30% fiber content [27]. Furthermore, the study highlights that these figures could become even more favorable as these biopolymers are effectively and widely incorporated into the market, in line with the conclusions published by Vandi et al. [28].

The published studies related to composites based on PHA and lignocellulosic fibers generally demonstrate a reinforcing effect of the fibers [29,30,31,32,33,34,35]. However, the overall performance is greatly influenced by the nature and structure of the fibers used, as well as the compatibility strategies employed, due to the limited interfacial adhesion. Moreover, the impact of these fibers on biodegradability is still not fully understood. Indeed, the documented results yield highly variable outcomes depending on the conditions and environments considered. In general, the reported results in soil [36,37,38,39,40,41] and marine [42,43] environments show accelerated biodegradability, however, in industrial composting conditions.

While in soil and marine environments, the reported results generally demonstrate an acceleration of biodegradation, the same does not occur under industrial composting conditions. Avella et al. [44] observed a reduction in the biodegradation rate for wheat straw contents exceeding 10% in compounds based on polyhydroxybutyrate (PHB) [44]. Lignocellulosic fibers are mainly composed of hemicellulose, cellulose, and lignin. While cellulose and hemicelluloses are easily biodegradable, lignin is highly resistant to microbial degradation. The typical pH and temperature conditions in a conventional composting process are not favorable for the growth of key lignin-degrading enzyme producers (white-rot fungi), which can hinder access to other fiber components, thereby slowing down the process [44,45,46]. However, other studies have shown a significant acceleration of biodegradation under composting conditions [47,48], which they attribute to the high hydrophilic character and structure of the fibers that promote their separation from the matrix, favoring the access of microorganisms.

Therefore, the impact of using lignocellulosic fibers depends on several factors, and an individualized study is essential to assess the suitability of a material to be safely introduced into organic recycling circuits, guaranteeing the quality of the resulting compost. Indeed, the European Composting Network (ECN) has expressed concern about the introduction of containers labeled as compostable in composting channels [49] since current regulations to assess their compostability (e.g., UNE-EN 13432:2001) contemplate very long biodisintegration and biodegradation times (up to 6 months), far from the usual composting cycles in currently existing facilities, which last from 10 to 16 weeks depending on the composting system used [50].

In this study, our goal is to investigate the potential of two agricultural waste-derived lignocellulosic fibers for the development of PHBV-based composites designed for short-term applications. We aim to assess the technical viability of these composites by examining their processability, mechanical and thermal performance, and compatibility with composting at the end of their intended usage.

The fibers used in this research include almond shell (AS) and Oryzite^®^ (OR). It is important to note that Oryzite^®^ is a patented material primarily composed of rice husk fibers obtained from the waste generated during white rice processing. This innovative material can be considered a precursor to polymer hybrid composites since it also incorporates glass microparticles as well as other additives to improve its rheological properties, as detailed in its patent [51].

## 2. Materials and Methods

### 2.1. Materials

A commercial grade Poly(3-hydroxybutyrate-co-3-hydroxyvalerate) (PHBV) with 3% hydroxyvalerate content was purchased from Naturplast (Ifs, France) in pellet form (PHI002). According to the literature, this grade has a molecular weight measured by size exclusion chromatography of 316 kDa [52]. Micronized almond shell (AS) was kindly supplied by Unió Corporació Alimentària (Reus, Spain). A masterbatch of PHBV with 30% of Oryzite^®^ RYZ100 was supplied by Cámara Arrossera del Montsià SCCL (Tarragona, Spain).

### 2.2. Composites Preparation

Before extrusion, PHBV and PHBV/Oryzite^®^ masterbatch were dried at 70 °C for at least 12 h in a Piovan DPA 10 (Santa Maria di Sala, Italy). AS was sieved through a 140 µm mesh and dried at 60 °C in an oven for (at least) 12 h.

Neat PHBV and PHBV/Fiber composites of 10, 20, and 30 wt% fiber content were prepared by extrusion in a single-screw extruder equipped with a Maddock screw and L/D ratio = 25 (Rheomix 3000P, ThermoHaake, Karlsruhe, Germany). The flat nozzle was coupled with a calendrer to obtain sheets of c.a. 200 µm nominal thickness.

The temperature profile in the extruder of neat PHBV and PHBV/AS composite used from hopper to nozzle was 150/160/173/170 °C, and the rotation speed was 80 rpm. The temperature profile in the extruder of samples of PHBV/Oryzite^®^ was 160/169/171/170 °C, and the rotation speed was 70 rpm. All the samples were manually premixed before extrusion and fed to the main hopper by an extruder feeder.

The nomenclature used for naming the different formulations studied is PHBV, AS-X for the composites with almond shell, and OR-X for the composites with Oryzite^®^, where X corresponds to the fiber content. Table 1 summarizes the samples studied and their compositions.

### 2.3. Morphology Characterization

The morphology of the PHBV, AS-X, and OR-X composites was studied by scanning electron microscopy (SEM) using a high-resolution field-emission JEOL 7001F microscope (Tokyo, Japan). Since PHBV displays significant susceptibility to thermal degradation, when subjected to SEM analysis, this can result in surface degradation, potentially leading to misinterpretation of the acquired images, especially in areas of heightened sensitivity, like interfacial regions. To mitigate this effect, real-time observations were performed, minimizing the exposure time of the sample. Furthermore, a low acceleration electron voltage of 5 keV was chosen to avoid any thermal degradation, enhancing the integrity of the analysis. Samples were fractured in liquid nitrogen and coated by sputtering with a thin layer of platinum prior to SEM observation. The morphology and dimensions of the fibers were also analyzed by SEM. The particle size distribution of the fibers was evaluated with the aid of the Fiji^®^ software (ImageJ 1.51j8) from 150× magnification SEM images [53]. Measurements were conducted individually for each particle in every picture without using automatic contrast filters.

### 2.4. Thermal Characterization

The thermal degradability of the composites was studied via TGA (Mettler Toledo, Barcelona, Spain) using a TG-STDA Mettler Toledo model TGA/SDTA851e/LF/1600. The composites were heated from 40 to 900 °C at a heating rate of 10 °C/min under nitrogen flow. The initial decomposition temperature (T_5%_ at 5% weight loss) was determined from the weight loss curve, and the maximum degradation rate temperature (T_d_) was measured at the derivate thermogravimetric analysis (DTG) peak maximum.

Differential scanning calorimetry (DSC) (Mettler Toledo, Barcelona, Spain) experiments were performed on a DSC (Mettler Toledo) with an intracooler (Julabo FT900) calibrated with an Indium standard before use. The samples weighing between 4 and 6 mg were first heated from 25 °C to 190 °C at 10 °C/min and kept for 3 min at 190 °C, then cooled to −20 °C at 10 °C/min, kept for 3 min at −20 °C, and finally heated to 190 °C at 10 °C/min. Melting temperatures Tm and enthalpies (${∆H}_{m}$), as well as the crystallization temperatures Tc and enthalpies (${∆H}_{c}$), were calculated from the second heating and cooling curves, respectively. In order to assess the influence of the secondary phase on the PHBV’s ability to crystallize, PHBV crystallinity ($X_{c}$) was determined by applying the following expression (1):

(1) $$X_{c}=\frac{{∆H}_{m}}{{∆H}_{m}^{0}\times w_{PHBV}}\times 100$$

where ${∆H}_{m}$ (J/g) is the melting enthalpy of the polymer matrix, ${∆H}_{m}^{0}$ is the melting enthalpy of 100% crystalline PHB (146 J/g) [54], and $w_{PHBV}$ is the polymer weight fraction of PHBV in the blend

### 2.5. Mechanical Characterization

Mechanical characterization was conducted through tensile tests. Dumbbell-shaped samples were cut from the films in both the MD (machine direction) and TD (transversal direction). These tests were carried out using a universal testing machine (Shimadzu AGS-X 5000N, Kyoto, Japan) equipped with a 500 N load cell at room temperature with a crosshead speed of 10 mm/min. The samples were tested after 15 days of aging to allow secondary crystallization to occur, reflecting its impact on their mechanical performance.

Additionally, tear tests were performed in both the MD and TD directions using the same equipment in accordance with the UNE-EN ISO 6383-1:2015 standard [55], with a testing speed of 200 mm/min until fracture. From the resulting force vs. displacement curves, the tear strength was calculated as the average tear force per unit thickness. These tear tests were also conducted after 15 days of aging. All the samples were stored in a vacuum desiccator at ambient temperature until testing.

To determine statistical significance, mechanical properties data were subjected to analysis of variance (ANOVA) using Statgraphics Centurion XVI version 16.1.17 (Manugistics Corp., Rockville, MD, USA). Significant differences were determined using the least significant difference test (*p* < 0.05).

### 2.6. Thermoforming

Vacuum-assisted thermoforming was conducted in a pilot plant (SB 53c, Illig, Helmut Roegele, Germany) equipped with an infrared emitter heating device. The mold used was a cylindrical male measuring 55 × 15 mm (diameter ×heigth). The heater was set at 600 °C while the heating time was changed in order to control the temperature of the polymer sheet. The sheets were printed with a square grid pattern (2 × 2 mm) to track the deformations that take place during the molding process. The shape reproducibility and the thickness distribution of the molded specimen were evaluated using three parameters based on the deformed grid after the thermoforming process. The three parameters to be evaluated in the thermoformed specimens are the thickness, corners, and edges, which appear in this order in the results. Each one was classified as “good” (green color, tick mark), “intermediated” (blue color, wave sign), and “bad” (red color, cross sign). The assessment was made considering the protocol of previous work [18]. Photographs were taken for the record.

### 2.7. Disintegration

Disintegration tests under standard composting conditions (ISO 20200) [56] were carried out with samples of 25 × 25 mm obtained from the films. Solid synthetic waste was prepared in accordance with the stipulations of the standard. Active mature compost was obtained from Hermanos Aguado, S.L. (Toledo, Spain).

The water content of the mixture was adjusted to 55%. The samples were placed inside inert supports to simplify their extraction and allow the contact of the compost with the samples, then buried in compost bioreactors at 4–6 cm depth. Bioreactors were incubated at 58 °C. The aerobic conditions were guaranteed by mixing the synthetic waste periodically and adding water to the standard requirements. Four replicates of each sample were removed from the bioreactors at different composting times for analysis. Samples were washed with water and dried under vacuum at 40 °C until a constant mass was reached. The disintegration degree was calculated by normalizing the sample weight to the initial weight according to Equation (2):

(2) $$\%D=\frac{m_{i}-m_{f}}{m_{i}}\times 100$$

where m_i_ is the initial dry mass of the material and m_f_ is the dry mass of the material recovered at different incubation stages.

## 3. Results and Discussion

### 3.1. Morphology Characterization

**Figure 1:**
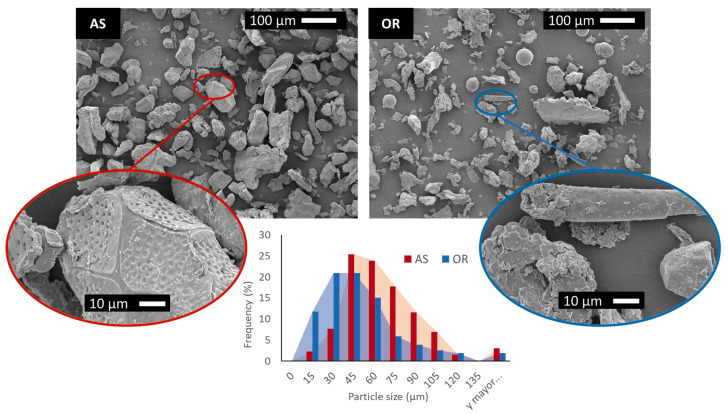
SEM results of AS and OR fibers morphology and their size distributions.

**Figure 2:**
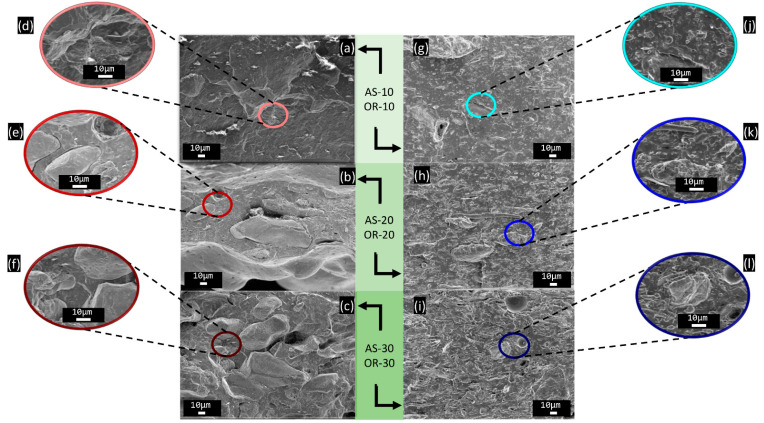
SEM results of PHBV/fiber composites: AS-10 micrographs at (**a**) 500× and (**d**) 1500×; AS-20 micrographs at (**b**) 500× and (**e**) 1500×; AS-30 micrographs at (**c**) 500× and (**f**) 1500×; OR-10 micrographs at (**g**) 500× and (**j**) 1500×; OR-20 micrographs at (**h**) 500× and (**k**) 1500×; OR-30 micrographs at (**i**) 500× and (**l**) 1500×.

The morphology of the fibers and composites was examined using SEM. Figure 1 and Figure 2 show representative micrographs of the fibers and their composites. This analysis was complemented with an image analysis from pictures at 150x magnifications to assess the average dimension of the fibers. The resulting measurements are reflected in the size distribution diagrams presented in Figure 1.

**Figure A1:**
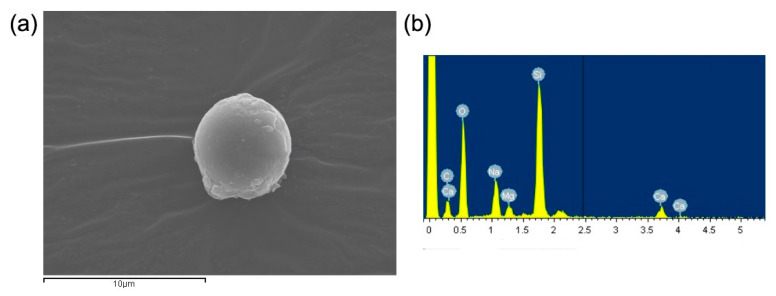
SEM microanalysis of (**a**) microbeads present in Orizyte^®^ and (**b**) elemental analysis of X-ray dispersive energy. This agrees with a soda–lime–silica glass.

The AS fibers exhibit a fairly regular, rounded morphology, with an average aspect ratio of 1.8 ± 0.7. The particle size distribution is quite narrow, with an average particle size of 58.7 µm. These particles are characterized by having a very porous surface, as can be observed in the magnified image of Figure 1. On the contrary, OR fibers are composed of particles with different shapes and aspect ratios ranging from 1 to 8, with an average value of 2.7 ± 1.5. The particle size distribution is broader than that corresponding to AS and is slightly shifted towards finer sizes, with a mean value of 43.7 µm. In general, the OR particles present a slightly rough surface but are much less porous than those of AS. The presence of spherical and smooth particles has been detected in OR. These particles correspond to glass microbeads, as confirmed by energy-dispersive X-ray elemental microanalysis (Figure A1).

As evidenced in Figure 2, the composites with the lowest AS content (AS-10) exhibit a uniform distribution of fibers within the PHBV matrix. However, as the fiber content increases, greater heterogeneity is evident, with noticeable fiber agglomerates. Furthermore, in AS-20 and AS-30 composites, the “pull-out” effect becomes apparent upon the freeze-fracture of the samples, which is not as clearly observed in AS-10 samples. In AS-10 samples, the fibers remain intact without being broken by the freeze-fracture process.

In contrast, in the composites containing Oryzite^®^, a uniform distribution of fibers is observed across the entire range of OR percentages studied. While some “pull-out” is observed in OR composites, the number of cavities and defects appears to be relatively low compared to AS composites. Nonetheless, the presence of a small gap in the interfacial region suggests a weak fiber–matrix interaction in both cases (AS and OR). Such a weak interfacial adhesion is consistent with the chemistry of both polymers and fibers, since lignin is more hydrophobic than polysaccharides, thus significantly limiting the extension of bond formation with the PHBV [57].

### 3.2. Thermal Characterization

#### 3.2.1. Thermogravimetric Analysis

**Figure 3:**
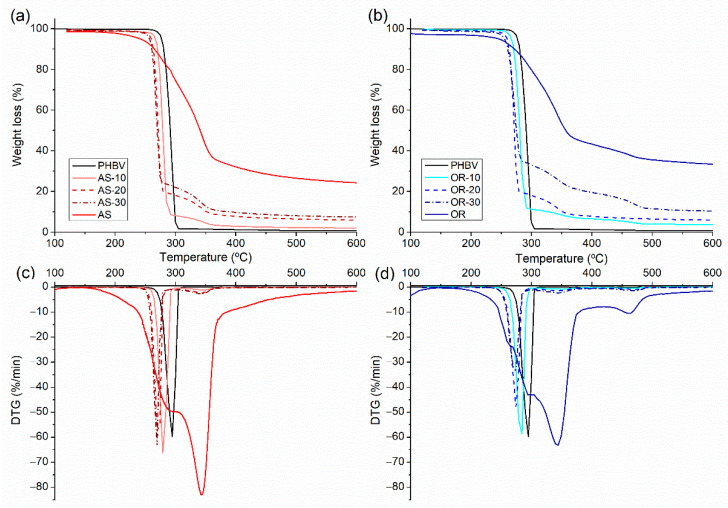
TGA(**a**) and DTG curves (**c**) of neat PHBV, AS, and PHBV/AS composites and the corresponding TGA (**b**) and DTG (**d**) curves for PHBV, OR and PHBV/OR composites.

Thermogravimetric analyses were performed after processing PHBV and their composites. AS and OR pristine fibers were also analyzed by TGA. Weight loss and DTG curves are shown in Figure 3. Table 2 summarizes the obtained parameters: the maximum degradation temperature of the polymer (T_d1_) and the maximum degradation temperature of the fibers (T_d2_), which correspond to the DTG peaks, and the onset degradation temperature, obtained from the weight loss curve (T_5%_) and the residue at 600 °C.

As widely reported, the thermal degradation of PHBV occurs abruptly in a single step through a random chain scission mechanism, with a maximum degradation temperature of approximately 296 °C [58,59]. In contrast, the thermal degradation of the fibers spans a wide temperature range (200–600 °C) [60]. This variance arises from the thermal degradation of the various components within the fibers, primarily composed of cellulose, hemicellulose, pectin, lignin, and waxes [61]. The thermal decomposition of pectin and hemicellulose primarily occurs in the range of 200–310 °C [62], while cellulose’s thermal degradation takes place in a single step, centered at 345 °C [63,64]. Lignin decomposition proceeds slowly, initiating around 200 °C and extending to temperatures close to 500 °C [64]. OR fibers exhibit a higher residue at 600 °C and an additional weight loss step at 460 °C compared to AS. The increased residue can be attributed to two factors: (i) the elevated silicon content in rice husk fibers, as widely reported [65,66], and (ii) the presence of the glass microspheres found in the SEM analysis of the Oryzite^®^ fibers. Moreover, the DTG peak observed at 460 °C may also be associated with the presence of processing additives in this commercial product.

In the case of composites, the first weight loss step can be attributed to the thermal degradation of PHBV, while the second and third (in the case of OR) correspond to the degradation of the fibers. The addition of fibers to the polymer leads to a reduction in both the onset and maximum degradation temperatures compared to neat PHBV. This reduction increases with the fiber content. The onset temperature reduction ranges from 10 °C for the lowest fiber content (10%) to about 20 °C for the highest fiber content (30%). The maximum degradation temperature is reduced by approximately 15°C for the lowest fiber content and 25 °C for the highest one. The thermal stability of PHBV is similarly affected by both types of fibers [10,47,67]. The residues at 600 °C of the PHBV/fiber composites are in accordance with the fiber content.

#### 3.2.2. Differential Scanning Calorimetry

**Figure 4:**
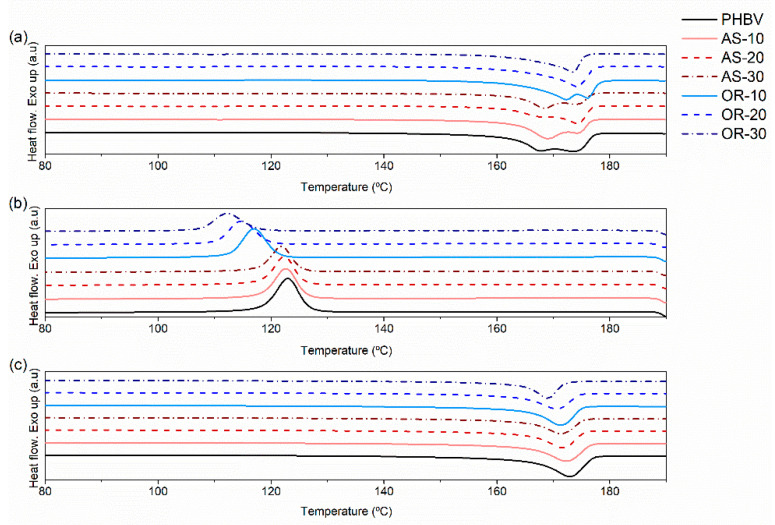
DSC thermograms of neat PHBV, AS, OR, and PHBV/AS and PHBV/OR composites: (**a**) first heating; (**b**) cooling; (**c**) second heating.

The influence of AS and OR on the melting and crystallization behavior of the composites was studied through DSC measurements. Figure 4 displays the DSC curves obtained from both heating and cooling scans. The key thermal parameters extracted from the thermograms are presented in Table 3.

In the first heating scans, neat PHBV, PHBV/AS composites, and OR-10 exhibit a double melting peak, whereas the inclusion of higher amounts of OR results in a single melting peak. The temperatures of the first part of a double-melting peak are denoted as T_m1_, and those corresponding to the second part of a double peak (or the peak of a single melting peak) are labeled as T_m2_. This double-melting peak phenomenon can be attributed to the presence of two populations of crystals with different crystallite lamellar thicknesses or degrees of crystalline perfection [68]. The first peak can be attributed to the fusion of less perfect and unstable crystals formed during the melt extrusion process, while the second peak corresponds to the fusion of more perfect and thermally stable crystals. During the DSC heating scan, there may also be a melting-recrystallization phenomenon involving the less-perfect crystals found in polyesters [69]. Notably, the double-melting peak phenomenon disappears after erasing the thermal history (second heating).

In Table 3, it can be seen that during the first heating scan, neat PHBV shows a crystallinity of 58.4%. However, the incorporation of fibers consistently diminishes PHBV’s crystallinity. Notably, in the case of PHBV/OR composites, an increase in fiber content results in a decrease in crystallinity, reaching as low as 47.5% with 30% of OR.

This influence of the fibers on crystallization is also observable during the cooling process. The crystallization enthalpy experiences a reduction from 89.4% to 61.3% (AS-30) and 53.5 (OR-30) with the incorporation of the highest content of fibers. Conversely, as the OR content increases up to 30%, the crystallization temperature of PHBV decreases from 123.9 to 112.5 °C, whereas it remains constant in the case of PHBV/AS composites.

It is interesting to note that after erasing thermal history and controlling cooling conditions, the double-melting peak phenomenon is eliminated. Also, whereas the melting temperatures (T_m_) of PHBV/AS composites are similar to those of PHBV, in the case of PHBV/OR composites, there is a slight reduction in T_m_, which becomes more pronounced with the increasing incorporation of OR fibers.

These findings suggest that the presence of fibers impedes the crystallization process of PHBV, particularly in the case of OR fibers, as previously noted in another study [10]. In contrast, the influence on final crystallinity is less pronounced with AS fibers.

### 3.3. Mechanical Characterization

**Figure 5:**
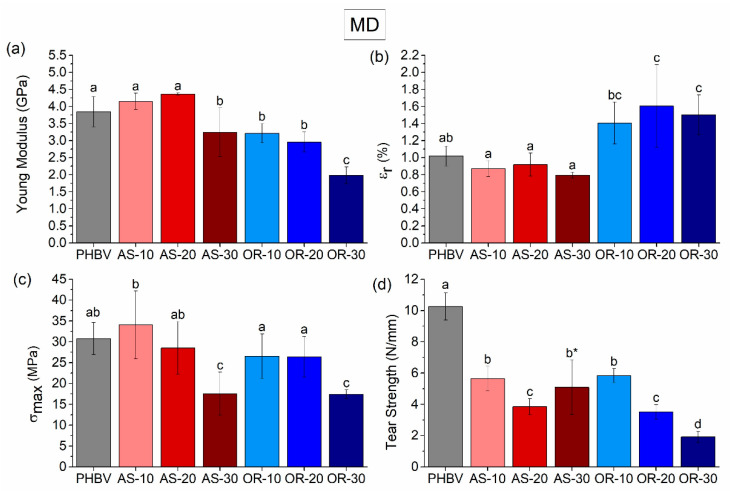
The tensile mechanical properties of the neat PHBV, the AS-X, and the OR-X composites in MD are (**a**) modulus of elasticity, (**b**) strain at break, (**c**) tensile strength, and (**d**) tear strength. Significant differences (*p* < 0.05) are indicated by different letters (a–d) above the bars. In Tear Strength, * above the bars indicates crack deviation during the test.

**Figure 6:**
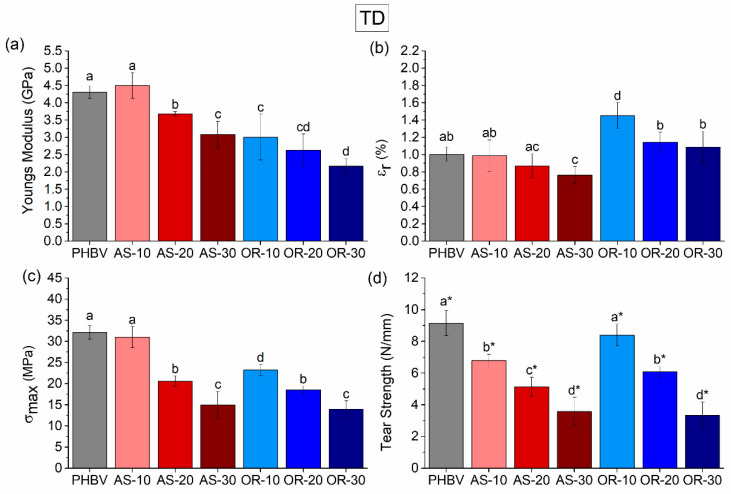
The tensile mechanical properties of the neat PHBV, the AS-x, and the OR-x composites in TD are (**a**) modulus of elasticity, (**b**) strain at break, (**c**) tensile strength, and (**d**) tear strength. Significant differences (*p* < 0.05) are indicated by different letters (a–d) above the bars. In Tear Strength, * above the bars indicates crack deviation during the test.

**Figure A2:**
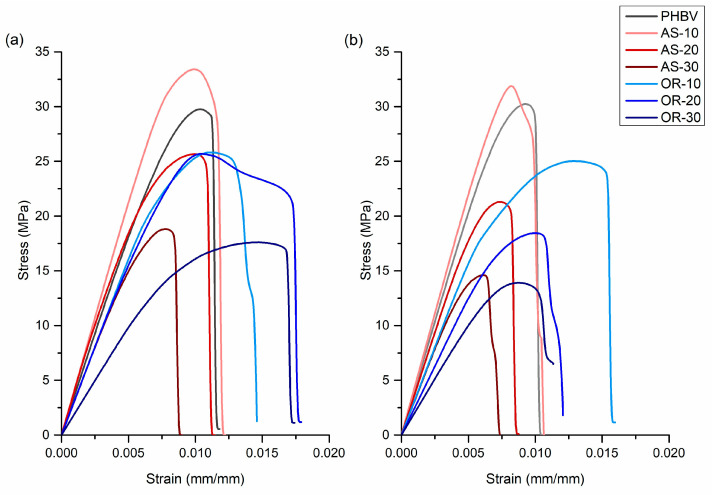
Representative stress vs. strain curves from tensile tests in (**a**) the MD direction and (**b**) the TD direction.

It is well known that PHBV can experience a combination of physical aging and a secondary crystallization phenomenon, which can impact its mechanical performance. Therefore, tensile tests were conducted after 15 days of aging at room conditions to allow for secondary crystallization to occur [70]. Indeed, the mechanical properties of the samples in the MD and TD were studied through tensile tests until failure, along with an assessment of their tear resistance. The properties obtained were compared in both directions, finding some differences when they were compared (see Table A1). Representative stress vs. strain curves are shown as Appendix A (Figure A2). Figure 5 and Figure 6 display the elastic modulus (E), tensile strength (σ_max_), strain at break (ε_r_), and tear strength in both the MD and TD directions.

In the MD (machine direction), a substantial decrease in the elastic modulus is evident in composites with the highest fiber content (AS-30 and OR-30) when compared to neat PHBV. Nevertheless, introducing AS content up to 20% has a minimal effect on the elastic modulus in this direction, while introducing an equivalent amount of OR fibers leads to approximately a one-third reduction. This trend is similarly observed in the TD (transversal direction), where differences in the elastic modulus become more pronounced with increasing either AS or OR content. Regarding tensile strength and tear strength, there is also a decrease with respect to neat PHBV as the fiber content increases (see Figure 5c,d for MD and Figure 6c,d for TD). Finally, there are no significant variations in strain at break (ε_r_) when AS is added, but there is an increase in this parameter if the OR is incorporated (Figure 5b and Figure 6b form MD and TD, respectively).

When comparing the trends found in modulus and tensile strength with other works, it can be noticed that these trends are the opposite of those found in other works, e.g., AS with PHB [31] or PHBV with Agave fibers [71]. Furthermore, it seems rather counterintuitive that adding reinforcement produces a decrease in modulus of elasticity and tensile strength. Indeed, by increasing the content of the fibers, the tear strength resistance of the compounds decreased. This behavior can be attributed to several factors: (a) there is weak interfacial interaction of the fibers with the polymeric matrix, as evidenced by the presence of “pull-out” defects in SEM analysis; (b) AS and OR fibers can act as stress raisers, promoting early failure at lower stresses [10]; and (c) the crystallinity of the PHBV matrix is also influenced by the amount of OR, as evidenced by the DSC thermograms.

The lack of a strong interface between the matrix and the fibers implies that the entire load during testing is supported by a matrix riddled with holes [72]. This phenomenon, along with the stress raised by the particles, can reduce the tensile strength, especially after the debonding of the second phase. In tear tests, this trend is more obvious since the fracture is localized at the notch and along the fractured area, not hindered by the bulk response of the material as it is in tensile tests.

On the other hand, it is worth mentioning that in OR composites, the elastic modulus decreases as the OR weight percentage increases. Also, the deformation at break increases by adding more OR. This behavior could be linked to the crystallinity of the samples. As indicated in Table 3, the crystallinity decreases with increasing fiber content, justifying the decrease in the elastic modulus. The reduced crystallinity allows for greater deformation of the material due to a higher amorphous fraction compared to neat PHBV and AS composites [73]. Furthermore, it is possible that certain rheological additives in the commercial Oryzite^®^ products [51] might contribute to this increase in the deformation at break value.

The tear tests also point to the effect of OR on the increase in deformability. In the case of the composites tested in the MD direction, as the fiber content increases, there is a reduction in tear resistance for all composites, as explained before, due to the stress-raising phenomenon attributed to the weakly bonded fibers. Interestingly, at a 30% fiber content, while the average tear resistance value for AS is higher than that of OR, AS composites experienced brittle fractures, whereas OR composites achieved complete tearing. These findings are consistent with OR composites offering improved ductility. Now, considering the TD direction, we see a similar trend with lower tear resistance as the fiber content increases. However, it is important to highlight that, in this direction, none of the samples managed to achieve complete tearing. Instead, crack deviation was observed in all cases, confirming an overall low toughness.

### 3.4. Thermoforming

**Figure 7:**
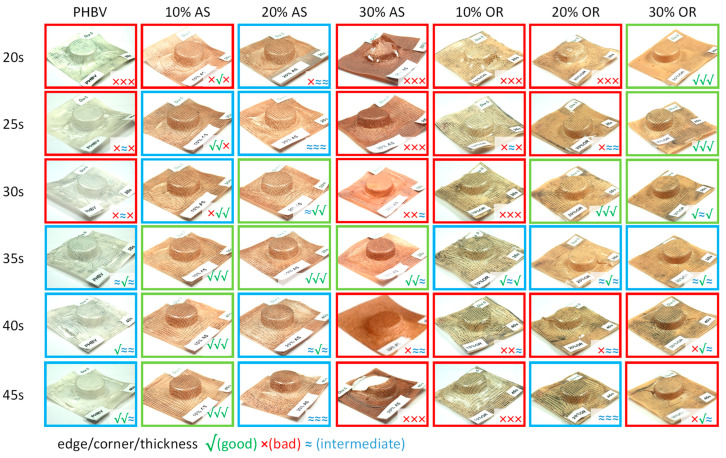
Photographs and assessments of the thermoforming ability of the trays show different heating times for neat PHBV, PHBV/AS composites, and PHBV/OR composites. The frame color indicates the overall quality of the thermoforming.

The thermoforming evaluation involved the visual inspection of the thermoformed trays, and the results are summarized in Figure 7, which also considers the heating time in the thermoforming machine.

As depicted in Figure 7, the inclusion of 10% and 20% AS into PHBV notably enhances the thermoforming process over various exposure times, as indicated by the increased presence of blue and green colors in the figure. This signifies a broader temperature processing window compared to pure PHBV. Specifically, for AS-10, the processing window ranges from 25 to 45 s of heating time, while for AS-20, it extends from 20 to 45 s. Both composites also demonstrate improved thickness distribution. However, when 30% AS is introduced to PHBV, the thermoforming process takes a negative turn compared to AS-10 and AS-20. This results in considerably poorer thickness distributions and a noticeable reduction in the processing window.

On the other hand, OR exhibits a different behavior compared to AS. Composites containing 30% OR display excellent performance in thermoforming trays, expanding the thermoforming time range from 20 to 35 s during heating. When 20% OR is added, the processing window becomes comparable to that of OR-30 composites. However, as the fiber content falls below 20%, the processing window decreases significantly and becomes more akin to pure PHBV than composites. In all three scenarios, there is an enhancement in thickness distribution when compared to PHBV, with the level of improvement increasing as the OR content rises.

### 3.5. Disintegration

**Figure 8:**
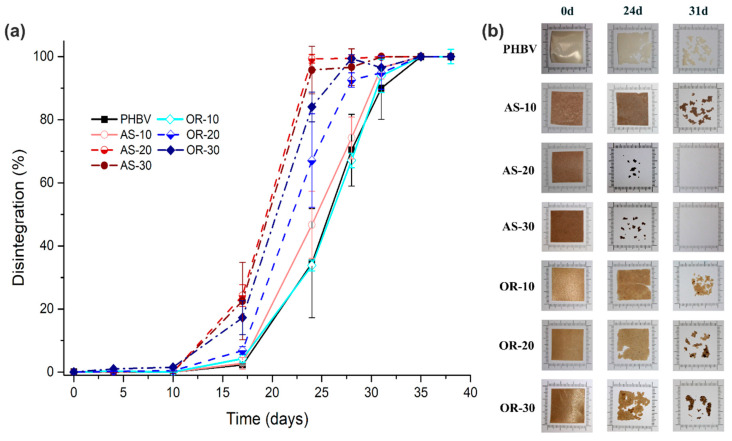
(**a**) Disintegration (%) rate in composting conditions as a function of time; (**b**) visual appearance of neat PHBV and its composites of AS and OR removed from the compost at different composting times.

The study on disintegration under composting conditions involved assessing the weight loss of neat PHBV and its composites in accordance with ISO 20200 standards. Figure 8 illustrates the disintegration rate of these samples over time, alongside the visual appearance of neat PHBV and its AS and OR composites, which were retrieved from the compost at various intervals.

The disintegration process typically initiates with an initial stage where enzymes are released, leading to the hydrolysis of the polymer matrix and the fragmentation of polymer chains. This process results in the formation of functional groups that enhance hydrophilicity and encourage the adhesion of microorganisms to the polymer matrix’s surface [74,75].

During the initial 10-day period, no noticeable weight loss occurs in the samples, indicating the presence of an induction period in the disintegration process. Starting from day 17 of composting, all samples exhibit weight loss, with AS-20 and AS-30 composites showing a more pronounced effect. By day 24, AS-20 and AS-30 composites nearly achieve complete disintegration, as visually evident in Figure 8b. However, AS-10 only reaches 47% disintegration, closely resembling the disintegration observed in pure PHBV [76]. Beyond day 31, AS-20 and AS-30 are entirely disintegrated, while AS-10 demonstrates a 96% disintegration rate, reaching complete disintegration before 35 days. The incorporation of 20% and 30% AS fibers into PHBV leads to a 30% reduction in the time required for complete disintegration compared to neat PHBV.

Regarding the OR composites, it is worth noting that on day 24, both OR-20 and OR-30 composites exhibit a notably higher disintegration rate when compared to OR-10, reaching disintegration levels of 64% and 87%, respectively. Interestingly, OR-10 disintegrates at a rate similar to pure PHBV. As we move to day 31, it becomes clear that, except for OR-10 and PHBV, all samples demonstrate a disintegration rate of over 95%, with complete disintegration achieved by all samples within 35 days of testing. These results align with earlier studies [31,77].

In summary, the introduction of both AS and OR fibers in higher concentrations fosters degradation in comparison to PHBV. This observation can be attributed to the limited interaction between the matrix and fibers, as indicated by scanning electron microscopy (SEM) analysis. The weak interfacial adhesion, combined with the fibers’ high moisture absorption capacity, facilitates their detachment from the matrix. This, in turn, improves water accessibility to the matrix and encourages microorganism adhesion to the sample surface, thus promoting hydrolysis, fragmentation of the composites, and their bioassimilation [43,45,48]. Similar findings have been reported in studies conducted by Wu [78], Oliveira et al. [79], and Ramos et al. [80], which investigated the biodegradation of biopolymers with various natural fibers. The weight loss resulting from this degradation is visually evident in Figure 8b. Notably, all samples undergo degradation within 35 days of testing, ensuring compliance with regulatory standards.

## 4. Conclusions

In conclusion, we successfully developed fully biodegradable biocomposites by incorporating micronized almond shell (AS) and Oryzite^®^ (OR) into PHBV, with fiber loadings of up to 30%. While the thermal stability of these composites slightly decreased, it remained sufficient for typical processing conditions.

Notably, the OR-based composites exhibited lower crystallinity and reduced crystallization temperatures compared to both PHBV and AS composites. However, when it comes to mechanical performance, the composites did not enhance the tensile properties of PHBV. This can be attributed to the weak interaction between the PHBV matrix and the fibers, as well as a lower crystallinity index.

Nevertheless, an important finding is that the incorporation of both AS and OR fibers extended the processing window compared to pure PHBV. These results offer a promising outlook for similar biocomposites when compatible with suitable sustainable additives in the future.

Moreover, incorporating significant proportions of AS substantially decreases disintegration times during composting, aligning them more closely with the timeframes typically seen in industrial organic recycling processes. Consequently, these compounds exhibit great potential to be integrated into biocircularity systems.

## Figures and Tables

**Table 1:** Nomenclature and composition of the samples.

Sample	PHBV ^1^ (wt%)	AS (wt%)	OR (wt%) [Masterbatch Content] ^2^
PHBV	100	0	- [-]
AS-10	90	10	- [-]
AS-20	80	20	- [-]
AS-30	70	30	- [-]
OR-10	90	-	10 [33]
OR-20	80	-	20 [66]
OR-30	70	-	30 [100]

^1^ Total PHBV wt% including PHBV from masterbatch. ^2^ PHBV/OR Masterbatch wt% used with pristine PHBV.

**Table 2:** TGA parameters.

Composition	T_5%_ (°C)	T_d1_ (°C)	T_d2_ (°C)	Residue at 600 °C (%)
PHBV	277	296	-	0.84
AS	242	-	344	24.28
AS-10	267	280	343	2.02
AS-20	259	271	344	5.92
AS-30	256	269	343	7.42
OR	245	-	343	33.43
OR-10	267	282	348	3.70
OR-20	257	275	347	6.59
OR-30	255	272	344	10.37

**Table 3:** DSC parameters.

	First Heating				Cooling		Second Heating		
Composition	ΔH_m_ (J/g)	T_m1_ (°C)	T_m2_ (°C)	Χ_c_ (%)	ΔH_c_ (J/g)	T_c_ (°C)	ΔH_m_ (J/g)	T_m_ (°C)	Χ_c_ (%)
PHBV	85.3	167.5	173.4	58.4	89.4	123.9	99.1	172.4	67.9
AS-10	70.3	168.8	173.9	53.5	77.4	123.2	86.6	171.9	65.9
AS-20	58.8	168.0	173.9	50.3	68.0	122.8	75.9	171.3	65.0
AS-30	56.0	168.2	174.0	54.8	61.3	122.2	67.5	171.2	66.1
OR-10	70.4	172.0	175.8	53.6	75.4	117.5	84.4	170.9	64.2
OR-20	61.4	-	173.7	52.5	64.7	115.1	73.1	170.1	62.6
OR-30	48.5	-	173.0	47.5	53.5	112.5	60.2	168.6	58.9

## Data Availability

The data presented in this study are available on request from the corresponding author.

## Appendix A

**Table A1:** Statistical analysis of the mechanical properties.

Composites	Young Modulus	Tensile Strenght	Elongation at Break
PHBV_MD	0.1022	0.6857	0.7875
PHBV_TD			
AS-10_MD	0.0355	0.5758	0.2666
AS-10_TD			
AS-20_MD	0.0365	0.0525	0.6745
AS-20_TD			
AS-30_MD	0.7042	0.3131	0.5635
AS-30_TD			
OR-10_MD	0.5451	0.0098	0.7504
OR-10_TD			
OR-20_MD	0.2065	0.0005	0.0924
OR-20_TD			
OR-30_MD	0.2411	0.0012	0.0140
OR-30_TD			

*p*-values obtained with ANOVA analysis are presented. If the *p*-value is greater than or equal to 0.05, there is no statistically significant difference between the mean values of the parameters analyzed between the data obtained in MD and TD of the different compositions, at a confidence level of 95.0%.
